# Revisiting the Role of Exercise Countermeasure on the Regulation of Energy Balance During Space Flight

**DOI:** 10.3389/fphys.2019.00321

**Published:** 2019-03-29

**Authors:** Claire Laurens, Chantal Simon, Joan Vernikos, Guillemette Gauquelin-Koch, Stéphane Blanc, Audrey Bergouignan

**Affiliations:** ^1^Université de Strasbourg, Centre National de la Recherche Scientifique, Institut Pluridisciplinaire Hubert Curien UMR 7178, Strasbourg, France; ^2^Centre National d’Etudes Spatiales, Paris, France; ^3^Carmen INSERM U1060, Laboratoire de Recherche en Cardiovasculaire, Métabolisme, Diabétologie et Nutrition, Université de Lyon, Lyon, France; ^4^Human Nutrition Research Centre of Rhône-Alpes, Hospices Civils de Lyon, Lyon, France; ^5^Thirdage Health, Culpeper, VA, United States; ^6^Anschutz Health and Wellness Center, Anschutz Medical Campus, Aurora, CO, United States; ^7^Division of Endocrinology, Metabolism and Diabetes, University of Colorado, Anschutz Medical Campus, Aurora, CO, United States

**Keywords:** energy intake, energy expenditure, appetite, resistive exercise, aerobic exercise, HIIT, microgravity, astronauts

## Abstract

A body mass loss has been consistently observed in astronauts. This loss is of medical concern since energy deficit can exacerbate some of the deleterious physiological changes observed during space flight including cardiovascular deconditioning, bone density, muscle mass and strength losses, impaired exercise capacity, and immune deficiency among others. These may jeopardize crew health and performance, a healthy return to Earth and mission’s overall success. In the context of planning for planetary exploration, achieving energy balance during long-term space flights becomes a research and operational priority. The regulation of energy balance and its components in current longer duration missions in space must be re-examined and fully understood. The purpose of this review is to summarize current understanding of how energy intake, energy expenditure, and hence energy balance are regulated in space compared to Earth. Data obtained in both actual and simulated microgravity thus far suggest that the obligatory exercise countermeasures program, rather than the microgravity *per se*, may be partly responsible for the chronic weight loss in space. Little is known of the energy intake, expenditure, and balance during the intense extravehicular activities which will become increasingly more frequent and difficult. The study of the impact of exercise on energy balance in space also provides further insights on lifestyle modalities such as intensity and frequency of exercise, metabolism, and the regulation of body weight on Earth, which is currently a topic of animated debate in the field of energy and obesity research. While not dismissing the significance of exercise as a countermeasure during space flight, data now challenge the current exercise countermeasure program promoted and adopted for many years by all the International Space Agencies. An alternative exercise approach that has a minimum impact on total energy expenditure in space, while preventing muscle mass loss and other physiological changes, is needed in order to better understand the in-flight regulation of energy balance and estimate daily energy requirements. A large body of data generated on Earth suggests that alternate approaches, such as high intensity interval training (HIIT), in combination or not with sessions of resistive exercise, might fulfill such needs.

## Introduction

Humans have been present in space for more than 50 years. Data generated during human spaceflights have demonstrated that the space environment, characterized by microgravity, 90-min light/dark cycles and confinement, impacts almost all physiological systems inducing a myriad of adaptive responses. These alterations of the body’s physiology can jeopardize crew health and performance and thereby impact the overall success of the mission with a healthy return to Earth. Responses to microgravity include fluid redistribution, reduced plasma volume, rapid loss of muscle mass and strength, a switch from oxidative type I toward glycolytic type II muscle fibers, cardiovascular deconditioning, impaired aerobic exercise capacity, bone loss, immune and metabolic alterations, as well as central nervous system effects (Aubert et al., 2016; Bergouignan et al., 2016; White et al., 2016; Lang et al., 2017).

**FIGURE 1:**
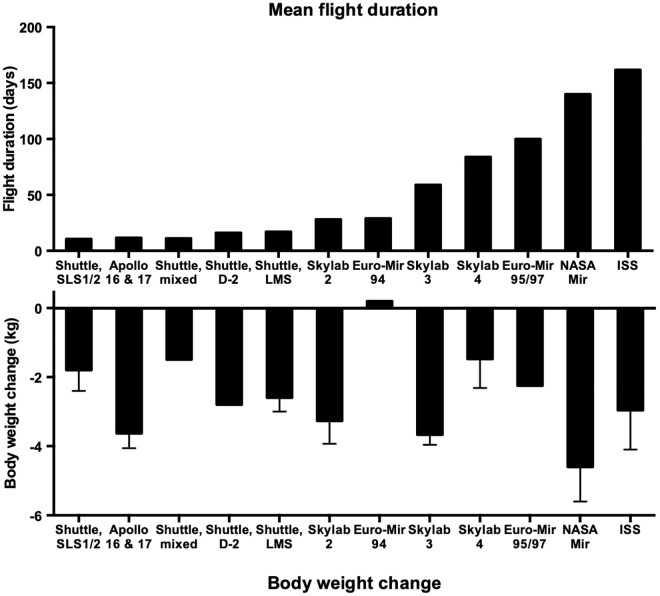
Body weight loss in space during past missions of different duration. Adapted from Wade et al. (2002) and Matsumoto et al. (2011).

Another common observation in most spaceflights was a systematic body mass loss independent of the space mission duration (Figure 1) (Wade et al., 2002; Matsumoto et al., 2011). On Mir (Smith et al., 1999, 2001), Shuttle (Stein et al., 1999a; Wade et al., 2002) and early ISS missions (Smith et al., 2005; Matsumoto et al., 2011), astronauts usually lost more than 5% of their pre-flight body mass. This was observed despite adequate food onboard (Lane and Schoeller, 1999). In many cases, this loss even exceeded 10%, which is clinically significant. However, body mass was successfully maintained stable in some missions like SLS1 and SLS2 in the 1970s (Thornton and Ord, 1975) or, more recently, on the ISS (Stein et al., 1996; Smith et al., 2005, 2012). Most recent reports on the ISS, however, show that astronauts still lose from 2 to 5% of their initial body mass during their time in space (Zwart et al., 2014). To prevent the loss of body mass was never a priority during the early missions, they were short and a moderate energy deficit is tolerable due to the availability of body fat stores. Concerns have been raised when missions targeting long stays in space in the context of planetary exploration were discussed. Beyond the simple loss of body weight, the energy deficit may have adverse health consequences over the long run.

Loss of body mass during spaceflight is associated with decreased muscle mass and functionality, incidence of cardiovascular issues, and oxidative stress (Stein, 2002; Smith and Zwart, 2008). Other human clinical studies and bed-rest studies conducted on Earth have demonstrated that a chronic energy deficit induces several deleterious consequences that are generally observed during space flight such as orthostatic intolerance, or may aggravate microgravity-induced adverse changes such as muscle loss, aerobic deconditioning, or disrupted immunity responses. This suggests that energy deficit may exacerbate the physiological adaptive responses to microgravity. Roadmaps establishing research priorities for planetary exploration raised the significance of energy balance regulation to a critical level, given it may represent an issue jeopardizing health and performance for long-duration missions (Stein, 2000; Bergouignan et al., 2016).

Although body water loss occurs during spaceflights (Zwart et al., 2014), it can only account for a portion of body mass loss. Muscle atrophy due to muscle disuse during space flights clearly contributes to body mass loss; however, it does not explain all of it. Body fat loss was indeed reported during in-flight experiments. For example, a decrease of about 2 kg in fat mass was observed following 2 weeks in space (Stein et al., 1999a). After the stabilization of fluid balance in the first few days in space, the body mass loss observed is therefore necessarily paralleled by a chronic alteration of energy balance: energy intake is not sufficient to match energy expenditure. Among the four studies published so far that investigated energy balance, three demonstrated a severe energy deficit during space flight (Rambaut and Leach, 1977; Stein et al., 1996, 1999a; Lane et al., 1997). During the 16-day LMS mission, by objectively measuring inflight double-labeled water-derived total energy expenditure, Stein demonstrated that energy expenditure was not matched by caloric intake. This mismatch led to an energy deficit as large as 5.7 ± 0.3 MJ/day in four astronauts (Stein et al., 1999a). If sustained, this may cause a weight loss of >5 kg/month. Recent developments of body mass measurement in space suggest that such a decrease in body mass may likely happen during the first month but then body mass would remain stable over the course of the mission (Zwart et al., 2014). However, when compiling all the available data on weight change during space missions (*n* = 619 missions), the extrapolation of weight loss via a linear model predicted that cumulative mean weight loss would be of 2.4% per 100 days (Matsumoto et al., 2011). A mission to Mars could therefore induce 15% loss of initial body mass or more, which could have serious health implications. Nevertheless, it is important to point out that all these studies were conducted with a very low sample size and a large inter-individual variability exists. This prevents drawing clear-cut conclusions but also emphasizes that energy balance regulation is a complex process.

Understanding how energy balance is regulated during space flight is key to prescribing the right amount of food to be consumed by the astronauts. A negative energy balance can be due to either insufficient energy intake, too high energy expenditure, or both. During space missions, an exercise countermeasure is commonly applied to astronauts to mitigate the microgravity-induced physiological adaptations, primarily muscle and bone alterations, and cardiovascular deconditioning. As currently prescribed, the exercise countermeasure program along with extravehicular activities impose a high volume of physical activity. This can be energetically costly and have a major influence on total energy expenditure. If food intake is not adjusted to match requirements, the high energy expenditure may lead to negative energy balance.

The purpose of this review is to summarize current understanding of the regulation of energy balance (i.e., energy intake and expenditure) in space and how the exercise countermeasure, as currently prescribed, affects this regulation. Based on the most recent evidence, we explain how the current exercise countermeasure program may be challenged, and what alternative approaches may be proposed for future space missions. Finally, we compare the impact of exercise on the regulation of energy balance in space and on Earth.

## Energy Intake and Anorexia During Spaceflights

A common observation in all manned spaceflight is that astronauts eat less than on Earth. They consume about 20–25% less calories than theoretically necessary to maintain a stable energy balance and therefore a stable body mass (Stein et al., 1999a, Heer et al., 2000; Stein, 2000; Smith et al., 2005; Zwart et al., 2014). However, this apparent reduction in food intake was derived as compared with the World Health Organization estimate of energy needs on Earth. Earth energy requirements may likely be different from energy needs in space. In addition, while calorie intake of astronauts on board the ISS has been slowly increasing during the last years, a large interindividual variability still exists (Zwart et al., 2014). Different factors may contribute to this uncoupling between energy intake and energy expenditure.

A first potential reason for reduced food intake is the poor attractiveness of the food available in space (Drummer et al., 2000; Cena et al., 2003). Even if food quality has been improved since the beginning of the space program thanks to partnerships with the food industry, the palatability of food provided to astronauts is still not comparable to what is available on Earth, and its diversity is limited. In addition, before the ISS, food habits, especially those related to cultural background, were not considered, which may have played a role in the lower food intake. International coordination imposed by the ISS has, however, slowly changed this aspect. The improvement in both variety and quality of food as well as awareness of cultural dietary preferences in space may have helped improve energy intake during recent missions. These changes may explain, at least partially, the reduction in body weight loss during the more recent ISS missions.

In addition to food intake, the type of food consumed and the amount of nutrients going through enterocytes and entering blood to be used by cells (i.e., metabolizable energy) can influence caloric intake. Astronauts are often susceptible in the first few days of the missions to space motion sickness, through neuro-vestibular mechanisms, which obviously acutely decreases food intake. This has subsequent modifications of gastric acid secretion, rhythmic contractions of stomach and intestine, and finally alteration of gastric emptying (Da Silva et al., 2002). The lower gastro-intestinal passage may also be driven by the microgravity-induced cardiovascular alterations that modify splanchnic blood flow, a physiological determinant of gastric emptying (Amidon et al., 1991). However, no direct measurements have been made during space missions. During a 20-day bed rest, we used a dietary tracer to assess changes in the kinetics of nutrient appearance in the blood as well as in nutrient extraction but did not notice any modification (Rudwill et al., 2018). This suggests that microgravity, at least under simulated conditions, does not modify the quantity of metabolizable energy. The macronutrient composition of the diet can also influence caloric intake. Astronauts have frequently reported a shift in dietary preference toward carbohydrates, which could play an independent role on energy intake (Bourland, 2000; Da Silva et al., 2002) Because carbohydrates are less energy dense than fats (4 versus 9 kcal/g), a diet relying upon a greater proportion of carbohydrate will provide less energy to the body. Another potential factor is the reduced thirst that is one of the early symptoms of space flight due to inoperant mechanisms underlying fluid and electrolyte balance (Vernikos, 1996).

The decrease in energy intake can also result from the loss of appetite that has been repeatedly reported during spaceflight. It may be due to both biological changes induced by microgravity and the influence of the space environment. The sensory responses to taste, smell, and texture of food (Drummer et al., 2000; Cena et al., 2003) are reduced, therefore leading to an alteration of food attractiveness/appeal. A number of additional satietogenic and orexigenic neurohormonal factors seem to be at play, directly or indirectly, during spaceflight. Anorexia during spaceflight and the potential underlying mechanisms have been fully reviewed by Da Silva et al. (2002). By compiling data from actual and simulated microgravity and human clinical studies and data from rodent models of simulated microgravity, they suggested that food intake and weight loss in the presence of microgravity are regulated by a complex repertoire of neuroendocrine and physiologic changes. In brief, concentration of leptin, a hormone secreted by adipose tissue that is known to increase satiety and reduce food intake, is increased during spaceflight (Stein et al., 1999b). A similar increase in plasma leptin concentration was observed under simulated microgravity conditions when adjusting for changes in fat mass (Bergouignan et al., 2010). In this same study, a rise in glucagon-like peptide-1 (GLP-1) plasma concentration, a satiety hormone produced by the intestine, was also observed, while ghrelin, the only orexigenic hormone known to date, was not modified (Bergouignan et al., 2010). Altogether these changes in appetite-related hormones may contribute to the reduced appetite observed during space flights. Other biological factors can influence appetite such as the preference of astronauts toward carbohydrates rather than fat. Greater carbohydrate intake has been associated with an increase in brain free-plasma tryptophan concentration, a precursor of the satietogenic serotonin neurotransmitter (Da Silva et al., 2002; Lam et al., 2010). The increase in astronaut body core temperature during spaceflight (Stahn et al., 2017) can be another one. Based on the “thermostatic theory of food intake” (Brobeck, 1948), high temperature has been associated with increased plasma concentration of the anorexigenic hormone polypeptide YY (PYY) and decreased relative energy intake in response to exercise (Shorten et al., 2009), while cold temperatures have been associated with greater food intake (Stroebele and De Castro, 2004). However, an inverse relationship has been reported between circadian core temperature variations and leptin concentrations (Simon et al., 1998), which rather suggests an association between lower physiological core temperature and satiety signals. Finally, stress experienced by the astronauts during spaceflights can alter both appetite and energy intake (Marti et al., 1994; Ans et al., 2018). Direct study of food intake, perceived appetite, changes in appetite-regulating hormones, and other physiological functions such as thermoregulation or digestion are clearly needed to provide insights on the biological mechanisms underlying the decrease in energy intake during space missions.

Space environment can also impact caloric intake. In a murine model of microgravity (i.e., hindlimb suspension), an increase in ambient CO_2_ concentration was shown to reduce food consumption (Wang and Wade, 2000). This suggests that the high concentration in CO_2_ present in the spacecrafts and the ISS may alter energy intake (Frey et al., 1998). It has also been suggested that light exposure continuously below threshold inside the spacecraft and intermittent 90-min day/night cycles in Earth orbit would be expected to affect eating behavior (Varma et al., 2000; Cena et al., 2003). Indeed, an alteration in the dark-and-light cycle disrupts circadian rhythm (Navara and Nelson, 2007; Wright et al., 2013) with consequences on hormone secretion and homeostasis (Kim et al., 2015). For example, alteration in light-dark cycle has been shown to alter the rhythmic secretion of leptin in rodents, and to impact food intake and body weight (Challet, 2017). On Earth, disruptions in circadian rhythms as well as sleep have been reported to alter the normal leptin 24-h pattern in humans (Simon et al., 1998). The continuous light astronauts are exposed to may therefore, at least partly, explain the greater leptin concentration observed in astronauts (Stein et al., 1999b), which may be involved in their development of loss of appetite during spaceflight (Varma et al., 2000). The influence of the space environment on appetite and energy intake warrants more research. However, it is important to note how challenging in-flight experiments are and the difficulty to properly control timing of measurements. Studies using simulated microgravity models on Earth may first be needed to obtain round the clock data on multiple parameters and disentangle these relationships. In this context, it is further important to accurately estimate the energy requirements in space.

## Energy Expenditure During Spaceflight and Simulated Weightlessness

When energy balance is stable, energy needs equal energy expenditure. Changes in total energy expenditure can result from modifications in one or more of these main components (Pinheiro Volp et al., 2011):

• Resting metabolic rate, energy necessary to maintain basal body functions at rest, thermoneutrality, and fasting;
• Diet-induced thermogenesis, energy allocated to the processing of ingested food (i.e., energy needed to process nutrients after a meal for use and storage);
• Energy cost of thermoregulation, energy allocated to maintain the body at steady temperature;
• Energy consumed in any body movement, which includes non-exercise (or daily life activities) and structured exercise energy expenditure. Physical activities, both spontaneous and structured or planned, can be of very low intensity including sedentary activities, low, moderate, and vigorous intensity. Intensity, duration, and frequency of the bouts of activity, define the volume of activity and impact energy expenditure related to physical activity. Physical activity energy expenditure is the most variable component of total energy expenditure.

Stein et al. (1999a) objectively measured inflight free-living total energy expenditure with the gold standard method of double-labeled water during the life and microgravity sciences (LMS) 16-day mission. He reported an energy expenditure of 0.9 ± 0.014 MJ/kg/day. He further estimated that total energy expenditure equals 1.4 × RMR + energy cost of structured physical exercise, where RMR is resting metabolic rate (MJ/d) and 1.4 an activity factor. This activity factor is the physical activity level defined as the ratio of total energy expenditure over resting metabolic rate. On Earth, it varies from 1.3 to 1.4 for sedentary and inactive individuals to 1.8–2.0 for sportive people, and can go up to 2.3–2.5 for world athletes (Westerterp, 2018). Non-exercising astronauts therefore have energy requirements close to sedentary and inactive adults in the general population but also to bed-rested participants. The 6° head-down tilt bed rest is a ground model of microgravity used on Earth to study physiological adaptations to weightlessness and test new countermeasures (Hargens and Vico, 2016). Using this model during a 42-day head-down tilt bed-rest study (Blanc et al., 1998), we estimated energy requirements for men to be 1.47 × RMR + energy cost of exercise. In another 2-month bed-rest studies, we further showed that energy requirements for women were 1.45 × RMR + energy cost of exercise (Bergouignan et al., 2010). Although resting metabolic rate has never been directly measured in space, data obtained during bed-rest studies showed a decrease in resting metabolic rate essentially due to fat-free mass loss from ambulatory conditions to bed rest (Blanc et al., 1998; Bergouignan et al., 2006, 2010). This reduction in metabolic rate, however, accounts for a somehow minor change in total energy expenditure, i.e., about 4% or 53 kCal in women (Bergouignan et al., 2010). Although no objective measurement has been reported in space, no change in diet-induced thermogenesis has been observed during bed-rest studies. It is, however, possible that a reduction in postprandial thermogenesis occurs in space, as foods are not consumed under the same solid nature and with the same nutritional density than on Earth. Indeed, a study conducted on Earth has demonstrated that the energy cost of food processing is higher after a solid compared to a liquid meal (Habas and Macdonald, 1998). At rest, the core temperature of the astronauts is 1°C above the core temperature measured on Earth (Stahn et al., 2017) probably because of the impaired convective heat transfer and evaporation process to cool down the body, low-grade pro-inflammatory responses to weightlessness, psychological stress-induced hyperthermia, and strenuous exercise protocols leading to this “space fever” (Fortney et al., 1998; Polyakov et al., 2001; Stahn et al., 2017). On Earth, body core temperature is positively correlated with energy expenditure (van Marken Lichtenbelt et al., 2002), but how changes in body core temperature influence 24 h energy expenditure in space is unknown. Based on all these data, it is probably the large decrease in physical activity due to muscle disuse that is responsible for the decrease in total energy expenditure. Altogether these data are showing that energy needs are similar on the ground for inactive individuals to those in space – the difference being mainly the energy cost of exercise. Why 24-h total energy expenditure is not matched by 24-h energy intake in space remains to be determined.

## Energy Expenditure, Rather Than Energy Intake, May Drive Body Weight Control in Space

To understand the regulation of energy balance in space, it is important to appreciate the coupling between energy expenditure and intake on Earth. A large body of data suggests that fat-free mass is a strong determinant of food intake (Blundell et al., 2012; Weise et al., 2014). This appears to be due to an increase in resting metabolic rate, as basal energy requirements are higher when fat-free mass is greater (Caudwell et al., 2013; Blundell et al., 2015a; Hopkins et al., 2016, 2018). Therefore, the decrease in fat-free mass due to muscle disuse consistently observed during spaceflight may partly explain the lower food intake of astronauts. However, this relationship seems to be more subtle and depends on energy status. While a positive relationship between fat-free mass and energy intake exists when individuals are at or close to stable energy balance, it is disrupted under energy imbalance situations (positive or negative). On Earth, in response to energy deficit, as it has been observed in astronauts, the associated decrease in fat-free mass and subsequently, resting metabolic rate, leads to an increase in energy intake rather than to a reduction, possibly as an attempt to restore fat-free mass (Dulloo et al., 2017; Stubbs et al., 2018). This compensatory effect is most likely true in the acute phase of energy deficit but as soon as a new steady-state in fat-free mass is reached the positive correlation between fat-free mass and energy intake may be reestablished. Over the long term, the lower fat-free mass in astronauts may therefore lead to lower energy intake. This scenario would need to be confirmed in space, but would explain the decrease in energy intake. The problem is that the decrease in energy intake is more pronounced than the reduction in the energy requirements leading to body mass loss.

**FIGURE 2:**
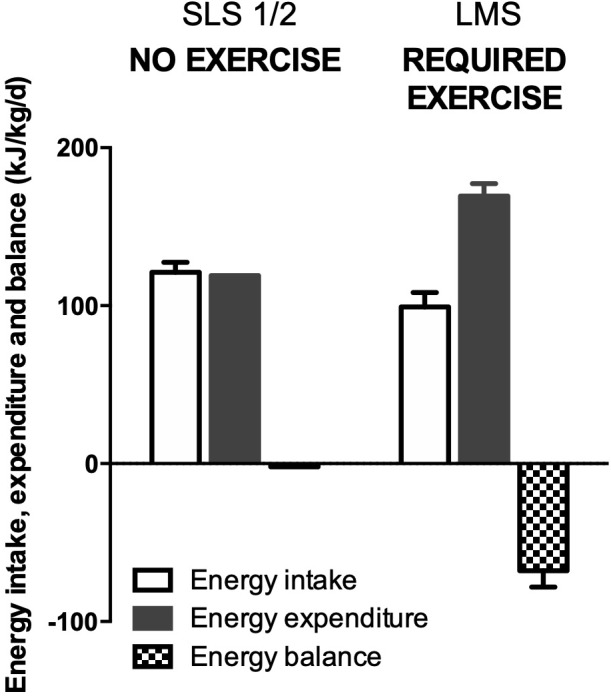
Energy intake, energy expenditure, and energy balance measured in space missions with or without exercise countermeasure. Adapted from Stein (2000).

**FIGURE 3:**
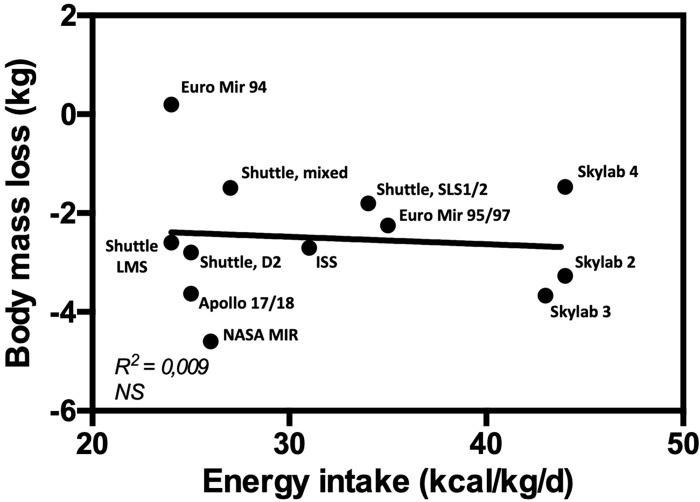
Correlation between body mass loss and energy intake.

**FIGURE 4:**
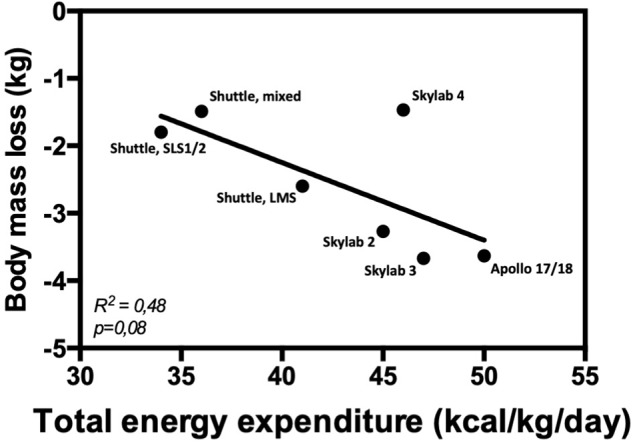
Correlation between body mass loss and total energy expenditure.

A recent elegant clinical study on Earth has suggested that 24-h energy expenditure, rather than resting metabolic rate or fat-free mass, is the main predictor of energy intake (Piaggi et al., 2015). In space, Stein (2000) has shown that during a mission in which no exercise countermeasure was required, energy intake matched energy expenditure thus keeping energy balance stable. The nitrogen balance was also stable indicating a maintenance of fat-free mass. Surprisingly, during a mission in which exercise was prescribed as a countermeasure against body deconditioning, energy intake was not matching energy expenditure leading to a negative energy balance (Figure 2). This was also associated with a negative nitrogen balance, suggesting a loss of muscle mass. Altogether, these data suggest that the increase in energy expenditure induced by physical exercise is not accompanied by compensatory changes in energy intake to match energy requirements, resulting in negative energy balance. This may further explain why there is no relationship between energy intake and body mass loss (Figure 3), but a positive association between total energy expenditure and total mass loss during spaceflights (Figure 4). When publishing these data, Stein (2000) suggested that the negative energy balance observed in presence of exercise was the result of the adverse effects of exercise on energy intake. They assumed that this negative feedback on energy intake was due to problems in disposing of the metabolic by-products produced during exercise. Given the impaired efficiency of the thermo-regulatory mechanisms in space, heat and CO_2_ produced during exercise would exacerbate the effects of higher body temperature, altered blood flow, and hypercapnia on appetite and energy intake. However, as we discussed it above, these factors may influence appetite but likely to a small extent. An alternative interpretation is that in space 24-h energy expenditure is the main driver of body mass changes, because energy intake cannot match it. This would be contrary to what is observed on the Earth where body weight loss is essentially reached by decreasing food intake (without spontaneous concomitant reduction in 24-h energy expenditure) rather than by increasing energy expenditure (Donnelly and Smith, 2005; Franz et al., 2007; Swift et al., 2018). Altogether these observations question the role of exercise in the regulation of energy balance in space and suggest that energy expenditure plays a key role in body mass control. These results further challenge the dogma of must-do exercise countermeasure, at least in its current form. To fully understand how exercise induces energy deficit in space, it is important to understand the respective impact of exercise on energy intake and expenditure, and the interaction between the different components of energy balance, both in space and on Earth.

## Role of Non-Exercise Activity Energy Expenditure in the Regulation of Energy Balance on Earth and in Space

Because physical activity is the most variable component of energy expenditure, it is thought that the greater moderate-to-vigorous intensity activity (including mainly exercise) is, the higher is total energy expenditure. However, this relationship may not be as straightforward. The different components of energy expenditure seem to interact with one another and play a role in the regulation of total energy expenditure and energy balance in a coordinated manner. The concept of an “activity-stat” was first introduced by TW Rowland in 1998. This concept suggests the existence of a biological control center regulating energy expenditure to a particular set point (Rowland, 1998). Since then, some evidence supporting this theory have been reported. Recently, Pontzer (2015) proposed the concept of constrained total energy expenditure in humans. Based on this concept, energy expenditure increases with physical activity at low activity levels but plateaus at higher activity levels as the body adapts to maintain total energy expenditure within a narrow range. The authors hypothesized that high activity levels may be offset by decreases in the other components of total energy expenditure, i.e., resting metabolic rate and diet-induced thermogenesis. This model has been used to explain why physical activity often has little impact on weight-loss strategies.

What is missing in this hypothesis is that, in addition to resting metabolic and diet-induced thermogenesis, activity energy expenditure can be subdivided in two other sub-components. It is composed of energy expended during structured exercise (i.e., exercise energy expenditure, ExEE, mostly related to moderate to very vigorous intensity activities) and non-exercise activities (i.e., non-exercise activity related energy expenditure, NExEE, including mostly light, and to some extent moderate intensity physical activities). On Earth, NExEE represents the energy expended in any body movement during daily life activities such as walking, taking the stairs, gardening, etc. Early measurement of energy expenditure in respiratory chambers indicated that NExEE accounts for a large amount of calorie expenditure even in sedentary individuals and may therefore constitute a buffering reservoir in case of exercise-induced increase in total energy expenditure (Ravussin et al., 1986). A recent review by Melanson (2017) compiled evidence indicating that NExEE decreases in both men and women during an exercise training program. We showed this is particularly true in overweight/obese individuals who spontaneously decrease NExEE in response to increased ExEE (Lefai et al., 2017). Therefore, rather than reductions in resting metabolic rate, the absence of increase in energy expenditure with structured physical activity is likely due to decreased NExEE and/or reductions in the energy cost of physical activity.

This regulatory process may, however, be different in space. Because of muscle disuse and reduced cost of locomotion in microgravity, the energy cost of daily activities is much lower, thus reducing NExEE. NExEE is, however, not null as indicated by the activity factor of 1.4 estimated by Stein et al. (1999a) in four non-exercising astronauts. This has two main implications. On the one hand, activity energy expenditure is mainly equivalent to the energy expended during exercise and extravehicular activity, and to a much lesser extent to NExEE. On the other hand, while engaging in the high volume of exercise prescribed as countermeasure, NExEE cannot be used to restore energy balance. Indeed, while the reduction in NExEE during space flights partly compensates for the increase in ExEE, this decrease is not sufficient to fully compensate for the high levels of the exercise prescribed as a countermeasure. This is because NExEE is the result of mandatory workload that cannot be ‘compressed’. Bed-rest studies have provided insights on the regulation of energy balance in microgravity conditions.

## Effect of Exercise on Total Energy Expenditure in Microgravity Conditions

**FIGURE 5:**
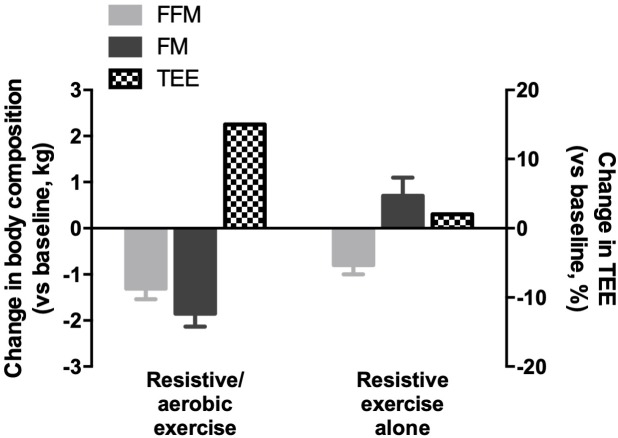
Effect of exercise countermeasure programs combining resistive and aerobic exercise versus resistive exercise alone on fat mass, fat-free mass, and total energy expenditure during bed-rest studies. FM: fat mass; FFM: fat-free mass; TEE: total energy expenditure.

Data we and others have obtained over the last 10 years during 10 to 90-day bed-rest studies provided some evidence supporting the hypothesis that exercise countermeasure may be the primary factor responsible for the negative energy balance during spaceflight (Bergouignan et al., 2010; Rudwill et al., 2013, 2015; Scott et al., 2014). Indeed, when subjects were fed either *ad libitum* or when energy intake was linked to requirements without exercise training, no significant change in fat mass was observed, reflecting a stable energy balance (Blanc et al., 1998). In contrast, when an exercise program combining resistive and aerobic exercise was performed, similar to the exercise countermeasure prescribed during spaceflights on the ISS, fat mass was strongly reduced reflecting a significant negative energy balance (Bergouignan et al., 2010) (Figure 5). In this study, resistance training was performed at maximal effort on a flywheel ergometer to train the thigh muscle groups using supine squat exercises during 35-min training sessions every 3 days. Aerobic training was performed three to four times per week with intensities varying from 40 to 80% of maximal oxygen uptake for 50 min. Importantly, despite this high volume of exercise and the combination of two types of exercise, this countermeasure protocol did not fully protect subjects from the loss of fat-free mass (Bergouignan et al., 2010). However, a training protocol based on resistive exercise only (flywheel ergometer, 35 min/day, 3–4 days/week) allowed to maintain fat-free mass at the baseline values during a 2-month bed rest in male subjects (Bergouignan et al., 2006), with no concomitant decrease in fat mass. Interestingly, while the resistive exercise protocol had a minor impact on total energy expenditure (+2%), the combined resistive and aerobic exercise countermeasure increased total energy expenditure by 15% (Bergouignan et al., 2010) (Figure 5). These data strongly suggest that energy expended during exercise is an important determinant of negative energy balance and weight loss during simulated microgravity in bed rest. Another important factor to consider is the cost of physical activity. Available data are conflicting. On the one hand, the cost of body movement is likely decreased in space compared to on Earth given the lower energy needed for weight-bearing muscle and muscle disuse. On the other hand, the only study conducted in space showed that the mechanical efficiency of walking and running on a treadmill was as low as 11% in space compared to 20% on Earth (Convertino, 1990), suggesting that the cost of activity is higher in space. These data have, however, to be considered with caution, as the subject loading during treadmill exercise is lower in space than on Earth. Oxygen consumption is therefore reduced, which is associated with a lower energy cost of exercise and/or metabolic efficiency. Another parameter to take into account is the metabolic efficiency that seems to be reduced, at least in simulated conditions (Greenleaf, 1989). Because the energy cost of exercise influences energy requirements, especially when large volumes of exercise and extra vehicular activities (EVAs) are prescribed, direct measurements of the efficiency of work and metabolism along with energy cost of activity are required to precisely evaluate the energy needs of astronauts.

**FIGURE 6:**
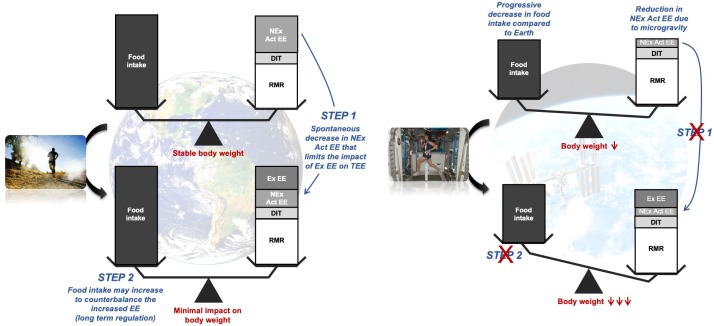
General model of energy balance regulation on Earth and in Space in response to exercise. EE: energy expenditure; TEE: total EE; NEx Act EE: non-exercise activity EE; Ex EE: exercise EE; DIT: diet-induced thermogenesis; RMR: resting metabolic rate.

Altogether these data support the hypothesis that a decrease in NExEE, rather than an increase in energy intake, is used primarily to buffer energy deficit induced by a high volume of exercise and represents a key component of energy balance control. Because this component is reduced in space due to the very nature of microgravity conditions, NExEE cannot be used (or only to a small extent) to restore energy balance when a high volume of exercise is prescribed as a countermeasure (Figure 6). The fact that energy intake cannot match energy expenditure when exercise is performed during spaceflight suggests a further negative feedback of exercise on appetite and energy intake.

## Effect of the Exercise Countermeasure on Feeding Behavior and Energy Intake

When energy balance was evaluated with a reference technique, as the difference between energy intake and total energy expenditure measured by the double-labeled water method during a 60-day bed-rest study, energy deficit was more pronounced in subjects following a protocol combining aerobic and resistive exercise than in the non-exercising group (Bergouignan et al., 2010). In this study, energy intake was calculated to counterbalance energy requirements, therefore maintaining a stable energy balance. Surprisingly, exercising subjects spontaneously reduced their energy intake below the prescribed values that accounted for the energy cost of exercise. This suggests that exercise impacts feeding behavior and hence food intake. Studies conducted on the ground have reported that aerobic exercise transiently suppresses appetite (Blundell et al., 2003; Broom et al., 2007), and this effect could be mediated, at least in part, by an increase in plasma satiety hormone PYY after aerobic exercise but not after resistance exercise (Broom et al., 2009). In addition, an increase in GLP-1 plasma concentration, a satiety hormone, has also been described after aerobic exercise sessions (Martins et al., 2007; Chanoine et al., 2008). A recent meta-analysis concluded that exercise induces a short-term energy deficit, as food intake is altered during the few hours following exercise (Schubert et al., 2013). It is possible that the increase in fat-free mass generated by resistive exercise is responsible for an increase in hunger to meet the increased energy requirements (Blundell et al., 2015b). In contrast, aerobic exercise has been associated with increased sensitivity of appetite control mechanisms (Blundell et al., 2015b). Clearly the influence of exercise on appetite is complex given it does not only depend on the type of exercise but also on the intensity. Contrasting results have indeed been published, suggesting that appetite-suppressing hormones are greater after high intensity exercise than after moderate-intensity aerobic exercise (Hazell et al., 2016). However, these studies mostly described effects of an acute exercise session and more work is clearly needed to assess the impact of changes in circulating anorexigenic hormone levels after exercise on subsequent food intake, and long-term appetite responses. In support of this, during a 60-day bed-rest study, combined aerobic and resistive exercise significantly increased fasting plasma GLP-1 concentration in women, which was associated with lower desire for food consumption. This later was itself positively associated with changes in energy intake (Bergouignan et al., 2010).

These data obtained in clinical and bed-rest studies are showing that exercise affects feeding behavior with an acute anorexic phenomenon which could exaggerate the loss of appetite and thus the negative energy balance. However, on Earth, over time, energy intake increases in response to greater energy expenditure induced by exercise training (Westerterp et al., 1992; Charlot and Chapelot, 2013). Most recent data obtained on the ISS suggest it may be the same in space. In addition, inter-individual variability may occur regarding the ability of astronauts to compensate the energy deficit. For example, Stein et al. showed that astronauts who were the most fit were those who experienced the most pronounced deconditioning and lost more weight (Matsumoto et al., 2011; Stein, 2013). Regardless of the time frame needed to adjust energy intake to energy expenditure, all these findings are important to consider when defining future exercise countermeasures, and an exercise countermeasure with minimum impact on appetite should be favored.

## Where Do We Go From Here?

A striking point when reviewing all this evidence is the fact that the importance of exercise during spaceflight has never been assessed under actual microgravity conditions. No trial has never been performed to rigorously evaluate the effect of the exercise countermeasure in space. This is an important point, as a large inter-individual variability in body weight loss has been observed and seems to be related to the energy cost of the exercise countermeasure. Furthermore, the adaptations of the body to microgravity *per se* (i.e., without any exercise countermeasure) remain a fundamental scientific question. This has been studied in bed-rest protocols on Earth, but the regulation of energy balance in simulated and actual microgravity is likely to be different. Studying the physiological impact of spaceflight without exercise countermeasure (extravehicular activities will obviously have to be sustained) would, however, provide with crucial information on whether, when, and how the body actually adapts to the space environment. Such a research study, however, raises important concerns; would it be ethical to ask astronauts to not perform exercise during spaceflight? A potential approach would be to run the study during the first period of the space flight, and instruct the astronauts to perform exercise during the last months of the mission in order to restore muscle and cardiovascular fitness before astronauts return to Earth. Although we believe this fundamental research question is important, we do not recommend against exercise countermeasure, but rather to rethink it.

As we reviewed it here, exercise, as currently prescribed, can negatively impact energy balance and other physiological systems. While exercise was likely of particular importance during early spaceflights to re-establish a certain level of physical activity, it may be less important for modern space missions during which astronauts are highly solicited for all type of tasks that involve more body movements. Therefore, we recommend that future studies evaluate energy requirements in astronauts and the energy cost of exercise in space as well as of the intense EVA. In order to better understand the in-flight regulation of energy balance and estimate daily energy requirements, the contribution of different components of energy balance (i.e., energy intake, total energy expenditure, ExEE, NExEE, resting metabolic rate, and diet-induced thermogenesis) should be evaluated along with the energy cost of activity. Because of its complexity, understanding how energy balance is regulated in space is of paramount importance to the health of astronauts and their mission success in future long-term missions. It will also be important from an economic and practical point of view considering the high cost of food transport and storage in a space station. For an individual estimated total energy expenditure of 12 MJ/day, a crew of six astronauts on a 3-year mission to Mars would require about 22 T of hydrated food, excluding water and the extra energy cost of exercise (Schoeller and Gretebeck, 2000). Even if water is to be totally recycled and food partially dehydrated, the cost of transport would be tremendously significant (20,000 Euros per kilogram), emphasizing the need to derive accurate energy requirements. On the other hand, a small underestimation of the energy requirements could impact health and survival of the astronauts.

Altogether, these data support the need to renew the discussion of the current exercise countermeasure program, especially in the context of planning for planetary exploration. Exercise countermeasures that effectively prevent body deconditioning from microgravity while having a limited effect on total energy expenditure needs to be developed. Others have previously raised this issue and started to test different exercise types to address this question (Matsuo et al., 2012). We specifically propose the use of an exercise countermeasure program that has minimum impact on total energy expenditure, that does not induce a loss of appetite and a reduction in energy intake, while benefiting muscle mass, protecting from muscle fiber type switch, preserving bone mass, intermediary metabolism, cardiovascular function, and aerobic fitness. Among the possibilities, resistive exercise could help prevent muscle mass and function without inducing a large increment on energy expenditure. However, its effect on the cardiovascular system would not be expected to be adequate in counteracting cardiovascular deconditioning (Belin de Chantemele et al., 2004). Interestingly, a large body of data generated on Earth shows that high intensity interval training (HIIT) fulfills all these needs (Alves et al., 2017; Winding et al., 2018). The benefits of HIIT to cardiometabolic health have been extensively studied during the past decade and detailed in many reviews (Bird and Hawley, 2012; Gibala et al., 2012, 2014; MacInnis and Gibala, 2017; Wormgoor et al., 2017). Importantly, HIIT has been proven efficient in protecting bone (de Jong et al., 2004; Aboarrage et al., 2018) and skeletal muscle mass loss (Osawa et al., 2014; Bell et al., 2015; Garcia-Pinillos et al., 2017; Taylor et al., 2017), improving metabolic health (Roberts et al., 2013; Little et al., 2011, 2014; Jelleyman et al., 2015; Madsen et al., 2015; de Souza et al., 2017) and cardiovascular function (Sloth et al., 2013; Hussain et al., 2016; Hannan et al., 2018), sometimes to a greater extent than traditional endurance exercise training (Kessler et al., 2012; Milanovic et al., 2015; Ramos et al., 2015; Karlsen et al., 2017; MacInnis and Gibala, 2017; Xie et al., 2017; De Strijcker et al., 2018). In addition, HIIT has a lower impact on total energy expenditure than continuous aerobic exercise (Matsuo et al., 2012) and requires a lower time commitment than endurance training (Gibala, 2007; Gibala and McGee, 2008; Gillen et al., 2016), which represents a major advantage in the context of an astronaut’s busy schedule during a mission. Testing the efficacy of a HIIT program in combination or not with sessions of resistive exercise as a countermeasure for future space missions therefore appears to be warranted. It will also be important to evaluate the frequency at which exercise needs to be performed: should astronauts perform a single bout of exercise once a day, or would it be more effective to fragment exercise into smaller bouts throughout the day? Studies conducted on Earth have demonstrated that exercise-related health benefits, especially with respect to glucose control and insulin resistance, are improved when subjects exercise regularly throughout the day rather than when a single longer bout of exercise is performed (Healy et al., 2008; Peddie et al., 2013). Additionally, HIIT relies primarily upon carbohydrate as fuel rather than fat, which may partially limit the loss of fat mass (De Jong et al., 2019). Another point to address will be to identify the best time to exercise. Indeed, a large body of research has shown that circadian rhythms impact exercise efficiency on Earth (Winget et al., 1985; Carrier and Monk, 2000; Teo et al., 2011). As these rhythms are unsettled during spaceflight due to the 90-min day/night cycle, studies should be performed to evaluate the best timing for exercising in space. Perhaps, exercise should be administered during every 90-min day-night cycle? While planet exploration programs are preparing to be launched, we should therefore approach with caution. Our knowledge of the relationship of exercise on human health in space remains to be fully understood. We are therefore entering a very exciting phase in space science research.

## Author Contributions

CL and AB wrote the first draft of the manuscript. All the authors edited it, approved the final version of the review, and contributed to the ideas presented in it.

## Conflict of Interest Statement

The authors declare that the research was conducted in the absence of any commercial or financial relationships that could be construed as a potential conflict of interest.
